# A noise-reduction GWAS analysis implicates altered regulation of neurite outgrowth and guidance in autism

**DOI:** 10.1186/2040-2392-2-1

**Published:** 2011-01-19

**Authors:** John P Hussman, Ren-Hua Chung, Anthony J Griswold, James M Jaworski, Daria Salyakina, Deqiong Ma, Ioanna Konidari, Patrice L Whitehead, Jeffery M Vance, Eden R Martin, Michael L Cuccaro, John R Gilbert, Jonathan L Haines, Margaret A Pericak-Vance

**Affiliations:** 1 Hussman Foundation, Ellicott City, MD, USA; 2 John P. Hussman Institute for Human Genomics, University of Miami, 1501 NW 10th Avenue, Miami, FL 33136, USA; 3 Vanderbilt Center for Human Genetics Research, Vanderbilt University, Nashville, TN, USA

## Abstract

**Background:**

Genome-wide Association Studies (GWAS) have proved invaluable for the identification of disease susceptibility genes. However, the prioritization of candidate genes and regions for follow-up studies often proves difficult due to false-positive associations caused by statistical noise and multiple-testing. In order to address this issue, we propose the novel GWAS noise reduction (GWAS-NR) method as a way to increase the power to detect true associations in GWAS, particularly in complex diseases such as autism.

**Methods:**

GWAS-NR utilizes a linear filter to identify genomic regions demonstrating correlation among association signals in multiple datasets. We used computer simulations to assess the ability of GWAS-NR to detect association against the commonly used joint analysis and Fisher's methods. Furthermore, we applied GWAS-NR to a family-based autism GWAS of 597 families and a second existing autism GWAS of 696 families from the Autism Genetic Resource Exchange (AGRE) to arrive at a compendium of autism candidate genes. These genes were manually annotated and classified by a literature review and functional grouping in order to reveal biological pathways which might contribute to autism aetiology.

**Results:**

Computer simulations indicate that GWAS-NR achieves a significantly higher classification rate for true positive association signals than either the joint analysis or Fisher's methods and that it can also achieve this when there is imperfect marker overlap across datasets or when the closest disease-related polymorphism is not directly typed. In two autism datasets, GWAS-NR analysis resulted in 1535 significant linkage disequilibrium (LD) blocks overlapping 431 unique reference sequencing (RefSeq) genes. Moreover, we identified the nearest RefSeq gene to the non-gene overlapping LD blocks, producing a final candidate set of 860 genes. Functional categorization of these implicated genes indicates that a significant proportion of them cooperate in a coherent pathway that regulates the directional protrusion of axons and dendrites to their appropriate synaptic targets.

**Conclusions:**

As statistical noise is likely to particularly affect studies of complex disorders, where genetic heterogeneity or interaction between genes may confound the ability to detect association, GWAS-NR offers a powerful method for prioritizing regions for follow-up studies. Applying this method to autism datasets, GWAS-NR analysis indicates that a large subset of genes involved in the outgrowth and guidance of axons and dendrites is implicated in the aetiology of autism.

## Background

Genome-wide association studies (GWAS) have provided a powerful tool for identifying disease susceptibility genes. However, analysis of GWAS data has been focused on single-point tests, such as the traditional allele-based chi-squared test or the Cochran-Armitage Trend test [1], which proceed by testing each single nucleotide polymorphism (SNP) independently. As it is likely that the disease variants have not been directly genotyped in a GWAS, tests that account for multiple flanking SNPs in linkage disequilibrium (LD) with the disease variants may increase the power to detect association [2].

Several approaches have been proposed in order to test for association based on multiple markers, which include the haplotype-based approach [3-5] and the multivariate approach [6,7]. Akey *et al. *[8] used analytical approaches to demonstrate that multilocus haplotype tests can be more powerful than single-marker tests. For the multivariate approach, tests such as Hotelling's *T*^2 ^test are often used to account for multiple markers jointly [6,9]. Although statistical power can be increased by such multi-marker approaches, it is not a straightforward operation to select markers for testing. Including all markers in a gene or region may not be feasible since it greatly increases the degrees of freedom in the test, which can reduce the power.

Follow-up studies, such as fine mapping and sequencing, are necessary in order to validate association signals and they are also challenging [2]. Prioritization of genes or regions for follow-up studies is often decided by a threshold of *P*-values or ranking for significant markers [10,11]. However, many false positives can still exist in the markers classified as significant for follow-up as a result of statistical noise and genome-wide multiple testing. Joint and/or meta-analysis of GWAS data can achieve greater power if these data or *P*-values are available from different datasets. If *P*-values from individual and joint analyses are available, it is possible to further increase the power by assigning more weight to markers with replicated association signals in several datasets or to markers that have flanking markers with an association signal.

We propose the use of the GWAS noise reduction (GWAS-NR) approach which uses *P*-values from individual analyses, as well as joint analysis of multiple datasets, and which accounts for association signals from surrounding markers in LD. GWAS-NR is a novel approach to extending the power of GWAS studies to detect association. Noise reduction is achieved by applying a linear filter within a sliding window in order to identify genomic regions demonstrating correlated profiles of association across multiple datasets. As noise reduction (NR) techniques are widely used to boost signal identification in applications such as speech recognition, data transmission and image enhancement, we expect that GWAS-NR may complement other GWAS analysis methods in identifying candidate loci that may then be prioritized for follow-up analysis or analysed in the context of biological pathways.

Enhancing statistical power is particularly important in the study of complex diseases such as autism. There is overwhelming evidence from twin and family studies for a strong genetic component to autism, with estimates of heritability greater than 80% [12-14]. Autism is generally diagnosed before the age of 4, based on marked qualitative differences in social and communication skills, often accompanied by unusual patterns of behaviour (for example, repetitive, restricted, stereotyped) [15]. Altered sensitivity to sensory stimuli and difficulties of motor initiation and coordination also are frequently present. Identifying the underlying genes and characterizing the molecular mechanisms of autism will provide immensely useful guidance in the development of effective clinical interventions.

Numerous autism candidate genes have been reported based on association evidence, expression analysis, copy number variation (CNV), and cytogenetic screening. These genes involve processes including cell adhesion (NLGN3, NLGN4 [16], NRXN1 [17], CDH9/CDH10 [18,19]), axon guidance (SEMA5A [20]), synaptic scaffolding (SHANK2, DLGAP2 [21], SHANK3 [22]), phosphatidylinositol signalling (PTEN [23], PIK3CG [24]), cytoskeletal regulation (TSC1/TSC2 [24,25], EPAC2/RAPGEF4 [26], SYNGAP1 [21]), transcriptional regulation (MECP2 [27], EN2 [28]) and excitatory/inhibitory balance (GRIN2A [29], GABRA4, GABRB1 [30]). However, aside from rare mutations and 'syndromic' autism secondary to known genetic disorders, the identification of specific genetic mechanisms in autism has remained elusive.

Over the past decade, the vast majority of genetic studies of autism (both linkage and focused candidate gene studies) have failed to broadly replicate suspected genetic variations. For this reason, the assumption that autism is governed by strong and pervasive genetic variations has given way to the view that autism may involve numerous genetic variants, each having a small effect size at the population level. This may arise from common variations having small individual effects in a large number of individuals (the common disease-common variant [CDCV] hypothesis) or rare variations having large individual effects in smaller subsets of individuals (the rare variant [RV] hypothesis).

Given the potential genetic heterogeneity among individuals with autism and the likely involvement of numerous genes of small effect at the population level, we expected that the GWAS-NR could improve the power to identify candidate genes for follow-up analysis. We applied GWAS-NR to autism GWAS data from multiple sources and conducted simulation studies in order to compare the performance of GWAS-NR with traditional joint and meta-analysis approaches. These data demonstrate that GWAS-NR is a useful tool for prioritizing regions for follow-up studies such as next-generation sequencing.

## Methods

### GWAS-NR

The GWAS-NR algorithm produces a set of weighted *P*-values for use in prioritizing genomic regions for follow-up study. Roeder and Wasserman [31] characterize the statistical properties of such weighting approaches in GWAS, observing that informative weights can improve power substantially, while the loss in power is usually small even if the weights are uninformative. The GWAS-NR algorithm computes a weight at each locus based on the strength and correlation of association signals at surrounding markers and in multiple datasets, without relying on prior information or scientific hypotheses. The weights are applied to the *P*-values derived from joint analysis of the complete data and the resulting weighted *P*-values are then used to prioritize regions for follow-up analysis.

Noise reduction methods are frequently applied when extracting a common signal from multiple sensors. The filter used by GWAS-NR is similar to the method proposed by de Cheveigné and Simon [32] for sensor noise suppression in magneto- and electro-encephalograph recordings. Each sensor is projected onto the other sensors and the fitted values from these regressions are used in place of the original values. The fitted values of such regressions retain sources of interest that are common to multiple sensors. As the regression residuals are orthogonal to the fitted values, uncorrelated components are suppressed.

In a genomic context, the 'sensors' take the form of probit-transformed *P*-values derived from independent datasets, as well as *P*-values derived from joint analysis of the full dataset. The filter inherently highlights cross-validating associations, by preserving signals that jointly occur in a given genomic region and attenuating spikes that are not correlated across subsets of the data. However, GWAS-NR can achieve no advantage over simple joint analysis when an association signal is restricted to a single marker and flanking markers provide no supplementary information.

We estimate ordinary least-squares regressions of the form

$$Z_{ij}=\alpha_{jk}+\beta_{jk}Z_{ik}+v_{jk}$$

and compute projections

$$\hat{Z_{ij}}=\alpha_{jk}+\beta_{jk}Z_{ik}$$

where *Z*_*i *_and *Z*_*ik *_are the probits *Φ*^-1^(1 - *p*) of the *P*-values at locus *i *in two datasets *j *and *k*. *Φ*^-1^(⋅) denotes the inverse of the cumulative standard normal distribution. The estimates are computed within a centred sliding window of *w *markers and *β*_*jk *_are constrained to be nonnegative which sets $\hat{Z_{ij}}$ to the mean $\bar{Z_{ij}}$ in regions having zero or negative correlation across sensors. As *β*_*jk *_is driven by the covariance between probits in datasets *j *and *k*, probits that demonstrate positive local correlation will tend to be preserved, while probits demonstrating weak local correlation will be attenuated. One local regression is computed for each locus and is used to compute a single fitted value $\hat{Z_{ij}}$ for that locus. The same method is used to compute projections $\hat{Z_{ik}}$.

In order to capture association signals at adjacent loci in different datasets without estimating numerous parameters, the regressor at each locus is taken to be the probit of the lowest *P*-value among that locus and its two immediate neighbours. Quality control (QC) failure or different genotyping platforms can cause SNP genotypes to be missing in different datasets. Missing genotypes for a locus having no immediately flanking neighbours are assigned a probit of zero. The window width *w *is calculated as *w *= 2*h *+ 1, where *h *is the lag at which the autocorrelation of the probits declines below a pre-defined threshold. In practice, we estimate the autocorrelation profile for each series of probits and use the average value of *h *with an autocorrelation threshold of 0.20.

After computing the projections of ${\overset{\wedge }{Z}}_{j}$ and ${\overset{\wedge }{Z}}_{k}$, the resulting values are converted back to *P*-values and a set of filtered *P*-values is computed from these projections using Fisher's method. The same algorithm is executed again, this time using the probits of the filtered *P*-values and the *P*-values obtained from the joint association analysis of the complete data. The resulting Fisher *P*-values are then treated as weighting factors and are multiplied by the corresponding raw *P*-values from the joint analysis, producing a set of weighted *P*-values. To aid interpretation, we apply a monotonic transformation to these weighted *P*-values, placing them between 0 and 1 by fitting parameters of an extreme value distribution. The GWAS-NR algorithm was executed as a Matlab script.

### Simulations

Although noise reduction has been shown to be useful in other biomedical applications [32], understanding its properties for identifying the true positives in disease association studies is also important. We used computer simulations to compare the performance of GWAS-NR with the joint association in the presence of linkage (APL) analysis and Fisher's method under a variety of disease models. We used genomeSIMLA [33] to simulate LD structures based on the Affymetrix 5.0 chip and performed the sliding-window haplotype APL [34] test to measure association. Detailed descriptions for the simulation settings are provided in Additional File 1 and detailed haplotype configurations can be found in Additional File 2.

An important goal for the proposed approach is to help prioritize candidate regions for follow-up studies such as next-generation sequencing. Top regions or genes ranked by their *P*-values are often considered priority regions for follow-up studies. In order to investigate the proportion of true positives that occur in the top regions, we treated the association tests as binary classifiers. The markers were ranked by their *P*-values and markers that occurred in the top *k *ranking were classified as significant, where *k *was pre-specified as a cut-off threshold. The markers that were not in the top *k *ranking were classified as non-significant. We then compared the sensitivity and specificity of GWAS-NR with the joint and Fisher's tests. The sensitivity was calculated based on the proportion of the three markers associated with the disease that were correctly classified as significant. The specificity was calculated based on the proportion of markers not associated with the disease that were correctly classified as non-significant. The sensitivity and specificity were averaged over 1000 replicates.

### Ascertainment and sample description

We ascertained autism patients and their affected and unaffected family members through the Hussman Institute for Human Genomics (HIHG, University of Miami Miller School of Medicine, FL, USA), and the Vanderbilt Center for Human Genetics Research (CHGR, Vanderbilt University Medical Center, Tennessee, USA; UM/VU). Participating families were enrolled through a multi-site study of autism genetics and recruited via support groups, advertisements and clinical and educational settings. All participants and families were ascertained using a standard protocol. These protocols were approved by appropriate Institutional Review Boards. Written informed consent was obtained from parents, as well as from minors who were able to give informed consent; in individuals unable to give assent due to age or developmental problems, assent was obtained whenever possible.

The core inclusion criteria were as follows: (1) chronological age between 3 and 21 years of age; (2) presumptive clinical diagnosis of autism; and (3) expert clinical determination of autism diagnosis using Diagnostic and Statistical Manual of Mental Disorders (DSM)-IV criteria supported by the Autism Diagnostic Interview-Revised (ADI-R) in the majority of cases and all available clinical information. The ADI-R is a semi-structured diagnostic interview which provides diagnostic algorithms for classification of autism [35]. All ADI-R interviews were conducted by formally trained interviewers who have achieved reliability according to established methods. Thirty-eight individuals did not have an ADI-R and, for those cases, we implemented a best-estimate procedure to determine a final diagnosis using all available information from the research record and data from other assessment procedures. This information was reviewed by a clinical panel led by an experienced clinical psychologist and included two other psychologists and a paediatric medical geneticist - all of whom were experienced in autism. Following a review of case material, the panel discussed the case until a consensus diagnosis was obtained. Only those cases in which a consensus diagnosis of autism was reached were included. (4) The final criterion was a minimal developmental level of 18 months as determined by the Vineland Adaptive Behavior Scale (VABS) [36] or the VABS-II [37] or intelligence quotient equivalent >35. These minimal developmental levels assure that ADI-R results are valid and reduce the likelihood of including individuals with severe mental retardation only. We excluded participants with severe sensory problems (for example, visual impairment or hearing loss), significant motor impairments (for example, failure to sit by 12 months or walk by 24 months) or identified metabolic, genetic or progressive neurological disorders.

A total of 597 Caucasian families (707 individuals with autism) were genotyped at HIHG. This dataset consisted of 99 multiplex families (more than one affected individual) and 498 singleton (parent-child trio) families. A subset of these data had been previously reported [19]. In addition, GWAS data were obtained from the Autism Genetic Resource Exchange (AGRE) [35] as an additional dataset for analysis. The full AGRE dataset is publicly available and contains families with the full spectrum of autism spectrum disorders. From AGRE, we selected only families with one or more individuals diagnosed with autism (using DSM-IV and ADI-R); affected individuals with non-autism diagnosis within these families were excluded from the analysis. This resulted in a dataset of 696 multiplex families (1240 individuals with autism) from AGRE [35].

### Genotyping and quality control and population stratification

We extracted DNA for individuals from whole blood by using Puregene chemistry (QIAGEN, MD, USA). We performed genotyping using the Illumina Beadstation and the Illumina Infinium Human 1 M beadchip following the recommended protocol, only with a more stringent GenCall score threshold of 0.25. Genotyping efficiency was greater than 99%, and quality assurance was achieved by the inclusion of one CEPH control per 96-well plate that was genotyped multiple times. Technicians were blinded to affection status and quality-control samples. The AGRE data were genotyped using the Illumina HumanHap550 BeadChip with over 550,000 SNP markers. All samples and SNPs underwent stringent GWAS quality control measures as previously described in detail in Ma *et al. *[19].

Although population substructure does not cause a type I error in family-based association tests, multiple founder effects could result in a reduced power to detect an association in a heterogeneous disease such as autism. Thus, we conducted EIGENSTRAT [38] analysis on all parents from analysed families for evidence of population substructure using the overlapping SNPs genotyped in both the UM/VU and AGRE datasets. In order to ensure the most homogeneous groups for association screening and replication, we excluded all families with outliers defined by EIGENSTRAT [38] out of four standard deviations of principal components 1 and 2.

### Haplotype block definition

We used haplotype blocks to define regions of interest. Significant regions can be used for follow-up analysis such as next-generation sequencing. We applied the haplotype block definition method proposed by Gabriel *et al*. [39] to the UM/VU dataset. We performed GWAS-NR based on single-marker APL *P*-values from UM/VU, AGRE and joint tests. We also performed GWAS-NR on *P*-values obtained from sliding-window haplotype tests with a haplotype length of three markers for the UM/VU, AGRE and joint datasets. Since the true haplotype length is not known, we chose a fixed length of three markers across the genome and used GWAS-NR to sort out true signals from the *P*-values. Blocks containing the top 5000 markers, as ranked by the minimum values (MIN_NR) of the GWAS-NR *P*-values obtained from single-marker tests, and the GWAS-NR *P*-values obtained from tests of three-marker haplotypes, were selected for further analysis.

### Combined *P*-values for haplotype block scoring

In order to test for the significance of the haplotype blocks, we calculated the combined *P*-value for each block using a modified version of the Truncated Product Method (TPM) [40]. TPM has been shown to have correct type I error rates and more power than other methods combining *P*-values [40] under different simulation models. Briefly, a combined score was calculated from the markers in each block, based on the product of MIN_NR that were below a threshold of 0.05. We used the Monte Carlo algorithm [40] with a slight modification to test the significance of the combined score. Specifically, a correlation matrix was applied to account for correlation among *P*-values for the markers in the same block. The null hypothesis is that none of the markers in the haplotype block are associated with the disease. In order to simulate the null distribution for the combined score, we generated two correlated sets of *L *uniform numbers based on the correlation of 0.67 for CAPL and HAPL *P*-values, where *L *denotes the number of tests in the block. The minimum values were selected from each pair in the two sets, which resulted in a vector of *L *minimum values. Then the correlation matrix was applied to the vector of *L *minimum values and a null combined GWAS-NR score was calculated for the haplotype block.

### Functional analysis

In order to investigate functional relationships among genes in the candidate set, each candidate was manually annotated and cross-referenced, based on a review of current literature, with attention to common functions, directly interacting proteins and binding domains. Supplementary functional annotations were obtained using DAVID (The Database for Annotation, Visualization and Integrated Discovery) version 6.7 [41-43].

## Results

### Simulations

**Figure 1:**
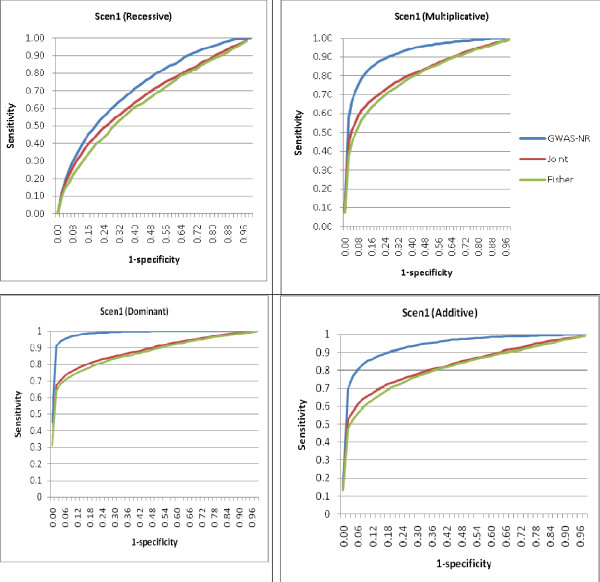
**Comparative classification rates for genome-wide association studies noise reduction (GWAS-NR), joint analysis and Fisher's test**. GWAS-NR has area under the curve (AUC) of 0.703 and the joint and Fisher's tests have AUC of 0.64 and 0.615, respectively, for the recessive model. Also GWAS-NR has AUC of 0.899 and the joint and Fisher's tests have AUC of 0.795 and 0.777, respectively, for the multiplicative model. For the dominant model, AUC for GWAS-NR, the joint and Fisher's tests are 0.981, 0.880 and 0.867, respectively. For the additive model, AUC for GWAS-NR, the joint and Fisher's tests are 0.932, 0.822, and 0.807, respectively.

**Figure 2:**
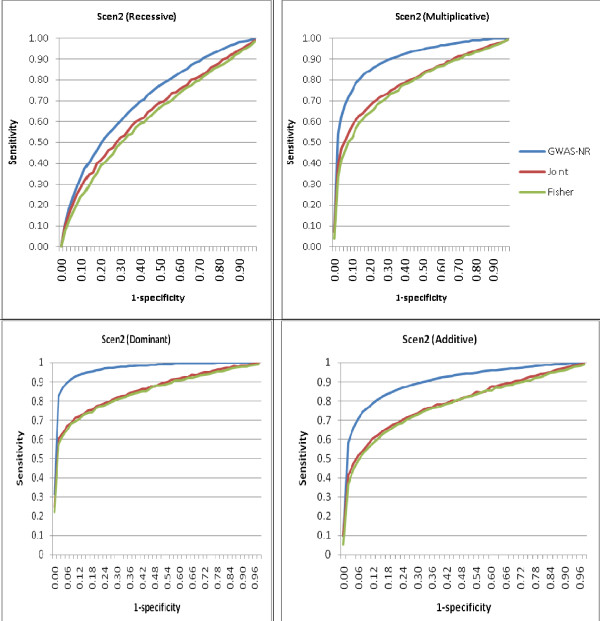
**Comparative classification rates for genome-wide association studies noise reduction noise reduction (GWAS-NR), joint analysis and Fisher's test with 20% and 50% missing markers**. GWAS-NR has area under the curve (AUC) of 0.689 and the joint and Fisher's tests have AUC of 0.622 and 0.598, respectively, for the recessive model. Also GWAS-NR has AUC of 0.883 and the joint and Fisher's tests have AUC of 0.776 and 0.760, respectively, for the multiplicative model. For the dominant model, AUC for GWAS-NR, the joint and Fisher's tests are 0.961, 0.852 and 0.844, respectively. For the additive model, AUC for GWAS-NR, the joint and Fisher's tests are 0.895, 0.785, and 0.775, respectively.

We present the simulation results for the three-marker haplotype disease models in Figures 1 and 2. Figure 1 presents receiver operating characteristic (ROC) curves to show the sensitivity and specificity of GWAS-NR, the joint APL analysis and Fisher's tests, based on varying cut-off values of ranking for significance. The Fisher's test to combine *P*-values was used here as a standard meta-analysis approach. The performance of a classification model can be judged based on the area under the ROC curve (AUC). For scenario 1 (identical marker coverage in each dataset), GWAS-NR produced a greater AUC than the joint and Fisher's tests. It can also be observed from the figure that, given the same specificity, GWAS-NR achieved a higher sensitivity for classifying true positives as significant as the joint and Fisher's tests.

As independent datasets may have an imperfect overlap of markers, which is true of the UM/VU and AGRE autism data, and the omission of the closest disease-related polymorphism from the data can have substantial negative impact on the power of GWAS [44], we also compared the performance of GWAS-NR with the joint APL tests and Fisher's tests under a range of missing marker scenarios: 20% of the simulated markers in one dataset were randomly omitted for the recessive and multiplicative models and 50% of the simulated markers were randomly omitted in one dataset for the dominant and additive models. This performance is shown in Figure 2. Again, the GWAS-NR produced a greater AUC than the joint and Fisher's tests and achieved a higher sensitivity for classifying true positives at each level of specificity.

The results for the two-marker haplotype disease models are shown in Additional File 3. The same pattern is also observed in Additional File 3 that GWAS-NR produced greater AUC than the joint and Fisher's tests.

We also evaluated the type I error rates of the modified TPM for identifying significant LD blocks using a truncation threshold of 0.05. For the scenario assuming full marker coverage as described in Additional File 1, the modified TPM had type I error rates of 0.035 and 0.004 at the significance levels of 0.05 and 0.01, respectively. For the missing-marker scenario, the type I error rates for the modified TPM were 0.046 and 0.007 at the significance levels of 0.05 and 0.01, respectively.

### Autism GWAS-NR results

We applied the GWAS-NR in autism data using UM/VU, AGRE and the joint (UM/VU)/AGRE datasets. A flow diagram (Additional File 4) for the data analysis process is found in the supplemental data. The selection of haplotype blocks based on Gabriel's definition resulted in a total of 2680 blocks based on the top 5000 markers. Moreover, 141 markers out of the 5000 markers which are not in any blocks were also selected. Blocks of LD were scored based on the truncated product of *P*-values below a threshold of 0.05 and a *P*-value for each block was obtained through Monte Carlo simulation. The *P*-values for 141 markers not in any blocks were also calculated using the Monte Carlo algorithm to account for the minimum statistics. All of the 141 markers had *P*-values less than 0.05 and were selected. 725 LD blocks achieved a significance threshold of *P *< = 0.01, and an additional 810 blocks achieved a threshold of *P *< = 0.05. A complete list of these blocks is presented in Additional File 5.

In order to determine what genes reside within the 1535 significant LD blocks, we used the University of California Santa Cruz (UCSC) Genome Browser Table Browser. The 1535 regions were converted into start and end positions based on the SNP positions in the March 2006 (NCBI36/hg18) human genome assembly. These start and end positions were used to define regions in the UCSC Table Browser. We searched each region for overlap with the RefSeq annotation track in the UCSC Browser. This search resulted in 431 unique genes which mapped back to 646 significant LD blocks and 50 single markers. These genes are presented in Additional File 6. For the remaining 839 LD blocks that did not overlap a RefSeq gene, we identified the nearest RefSeq gene using Galaxy [45]. The distance to these nearest genes averaged 417,377 bp with a range from 5296 to 5,547,466 bp. These nearest genes include candidate genes for which strong proximal associations with autism have previously been reported, such as CDH9 [18,19] and SEMA5A [20]. We considered these genes for follow-up because GWAS-NR, by construction, may capture association information from nearby regions that may not be in strict LD with a given SNP and because these proximal locations may also incorporate regulatory elements. These genes are presented in Additional File 7. Combining these sets resulted in a candidate set of 860 unique genes (presented in Additional File 8). For genes assigned to more than one significant LD block, the lowest *P*-value among these blocks is used for sorting and discussion purposes.

The most significant LD block we identified is located at 2p24.1 (ch2 204444539-20446116; *P *= 1.8E-06) proximal to PUM2. One LD block located within the PUM2 exon also had nominally significant association (*P *= 0.024). Additional top-ranking candidates, in order of significance, include CACNA1I (*P *= 1.8E-05), EDEM1 (*P *= 1.8E-05), DNER (*P *= 2.7E-05), A2BP1 (*P *= 3.6E-05), ZNF622 (*P *= 8.11E-05), SEMA4D (*P *= 9.09E-05) and CDH8 (*P *= 9.09E-05). Gene ontology classifications and InterPro binding domains reported by DAVID [41-43] to be most enriched in the candidate gene set are presented in Tables 1 and 2, respectively, providing a broad functional characterization of the candidate genes identified by the GWAS-NR in autism.

**Table 1:** Common functions of autism candidate genes identified by genome-wide association studies-noise reduction (GWAS-NR)

Gene ontology (GO) term	No. of genes	GO term identification	***P*-value**^**1**^	Examples
Cell adhesion	76	0007155	6.29E-13	CDH8, NCAM2

Biological adhesion	76	0022610	6.64E-13	CDH2, CTNNB1

Cell-cell adhesion	35	0016337	6.24E-08	CTNNA2, AMIGO2

Homophilic cell adhesion	21	0007156	1.21E-06	PTPRM, FAT1

Cell motion	44	0006928	6.65E-06	SEMA5A, FYN

Neuron differentiation	41	0030182	1.14E-05	EN2, NRXN1

Enzyme linked receptor protein signalling pathway	33	0007167	5.40E-05	NCK2, FGFR2

Neuron development	32	0048666	1.07E-04	ROBO2, RTN4R

Negative regulation of gene expression	42	0010629	1.27E-04	SIX3, CUX2

Axonogenesis	22	0007409	1.31E-04	SEMA6A, SLITRK5

Cell morphogenesis involved in differentiation	25	0000904	2.16E-04	PRKCA, PTK2

Cell motility	29	0048870	2.40E-04	DNER, PPAP2B

Localization of cell	29	0051674	2.40E-04	PTEN, NRP2

Negative regulation of transcription	38	0016481	3.19E-04	RBPJ, MEIS2

Cell morphogenesis involved in neuron differentiation	22	0048667	3.94E-04	PARD3, KALRN

Transmembrane receptor protein tyrosine kinase signalling	23	0007169	3.98E-04	SOCS2, DOK5

Neuron projection development	25	0031175	4.40E-04	RTN4R, NGF

Neuron projection morphogenesis	22	0048812	5.07E-04	PVRL1, CDH4

Regulation of cell projection organization	13	0031344	5.33E-04	SEMA4D, CDC42EP4

Negative regulation of nucleobase, nucleoside, nucleotide, and nucleic acid metabolic process	40	0045934	6.79E-04	BCL6, ZHX2

**Table 2:** Common binding domains of autism candidate genes identified by genome-wide association studies-noise reduction (GWAS-NR).

INTERPRO term	No. of genes	INTERPRO identification	***P*-value**^**1**^
Immunoglobulin I-set	20	IPR013098	8.97E-06

Cadherin	16	IPR002126	6.98E-05

Cadherin cytoplasmic region	7	IPR000233	1.14E-04

Pleckstrin homology	26	IPR001849	5.03E-04

Immunoglobulin	21	IPR013151	5.61E-04

Immunoglobulin subtype 2	21	IPR003598	6.77E-04

Fibronectin, type III-like fold	19	IPR008957	1.19E-03

Fibronectin, type III	19	IPR003961	1.72E-03

Epidermal growth factor (EGF)	14	IPR006209	3.71E-03

Meprin/A5-protein/PTPmu (MAM)	5	IPR000998	6.78E-03

Protein-tyrosine phosphatase, receptor/non-receptor type	7	IPR000242	7.36E-03

Pleckstrin homology-type	24	IPR001993	7.41E-03

von Willebrand factor, type A	10	IPR002035	7.41E-03

Immunoglobulin-like	35	IPR007110	7.57E-03

Cell adhesion represented the most common functional annotation reported for the candidate gene set, with a second set of common functional annotations relating to neuronal morphogenesis and motility, including axonogenesis and neuron projection development. Given the enrichment scores reported by DAVID [41-43] implicating neurite development and motility, and because numerous cell adhesion molecules are known to regulate axonal and dendritic projections [46,47], we examined the known functional roles of the individual candidate genes responsible for these enrichment scores. A total of 183 candidate genes were represented among the top 20 functional classifications reported by DAVID [41-43]. Based on annotations manually curated from a review of current literature, we observed that 76 (41.5%) of these genes have established roles in the regulation of neurite outgrowth and guidance. These include 39 (51.3%) of the candidate genes contained in the cell adhesion, biological adhesion, cell-cell adhesion and homophilic cell adhesion pathways.

Gene ontology [48] specifically associates two pathways with the narrow synonym 'neurite outgrowth': the neuron projection development (pathway 0031175); and the transmembrane receptor protein tyrosine kinase activity (pathway 0004714). To further test for functional enrichment of genes related to neurite outgrowth, we formed a restricted composite of these two pathways. Enrichment analysis using the EASE function of DAVID [41-43] rejected the hypothesis that this composite pathway is randomly associated with the autism candidate set (*P *= 2.07E-05).

Although many of the candidate genes identified by the GWAS-NR remain uncharacterized or have no known neurological function, we identified 125 genes within the full candidate set having established and interconnected roles in the regulation of neurite outgrowth and guidance. These genes are involved in diverse sub-processes including cell adhesion, axon guidance, phosphatidylinositol signalling, establishment of cell polarity, Rho-GTPase signalling, cytoskeletal regulation and transcription. Table 3 presents a summary of these genes by functional category. Additional File 9 presents annotations for these 125 candidates. Additional File 10 presents 104 additional candidates which have suggestive roles in neurite regulation based on putative biological function or homology to known neurite regulators but where we did not find evidence specific to neurite outgrowth and guidance in the current literature.

**Table 3:** Autism candidate genes with known roles in neurite outgrowth and guidance.

Function	Candidate gene (by lowest *P*-value)
Cadherin-catenin function	CDH8, CDH2, CDH11, CTNNB1, CTNNA2, PKP4, CTNND2, CDH4, CTNND1, CTNNA3

Cell adhesion	NCAM2, CNTN3, OPCML, ODZ4, NID1, CNTN5, F3, PVRL1, PTPRG, PARVA, FLRT2, ODZ2, NRXN1, ITGA9, ELMO1, FUT9, AMIGO2, KIRREL3, CNTNAP2, NTM

Ion channel	CACNA1I, CACNA1G

Axon guidance	SEMA4D, RTN4R, ROBO2, SEMA5A, PLXDC2, SLITRK5, SEMA6A, RGMA, UNC5D, ALCAM, NTNG2, RTN4RL1, PLXNC1, NRP2

Vesicle transport	STX2, STX16, STXBP5, SYT6

Post-synaptic scaffold	DLGAP2, MAGI1, MAGI2

Signal transduction	DNER, SPRY4, FRK, PRKCA, DOK6, PDE3A, FER, IRS2, SOCS2, SPRY2, FRS3, DOK5, FYN, LZTS1, PTPRD, FGFR2, NRG3, PPP2R2B ALK, RYR2, PALM2-AKAP2, MAP3K7, NTRK3, NGF, PPM1H, GDNF, CXCR4, PTK2, NEDD9, PTPN1, LEPR

Phosphatidylinositol signalling	PLA2G6, PIK3C2B, PTEN, PLA2G4A

Cell polarity	FAT1, PARD3, PARD6G, DCHS2

Rho-GTPase signalling	NCK2, DOCK1, PREX1, CDC42EP4, RND3, RGNEF, DOCK8, CIT, SRGAP3, KALRN, IQGAP2

Cytoskeletal regulation	SGK1, MYLK, GPR56, APBB1IP, PTPRM, WIPF3, PTPRT, MAP3K8, MICAL2, DGKG, COBL, CALD1

Transcription	PUM2, A2BP1, NKX6-1, SOX14, EN2, EBF1, MAP3K1, FOXG1, NFIC, BCL11A

Outside of functions relating to neuritogenesis, the most significant functional annotation reported by DAVID for the candidate gene set relates to transmission of nerve impulses (p = 9.02E-04). We identified 40 genes in the candidate set related to neurotransmission (synaptogenesis, neuronal excitability, synaptic plasticity, and vesicle exocytosis) which did not have overlapping roles in neurite regulation. Table 4 presents a summary of these genes by functional category.

**Table 4:** Autism candidate genes with roles in synaptic function.

Function	Candidate gene (by lowest *P*-value)
Synaptogenesis	LRRTM4, SYN3

Excitatory/inhibitory balance	KCNIP1, KCNQ1, KCNQ5, KCNJ4, SLC6A13, IQCF1, GABBR2, GRIK4, OAT, KCNN3, GRM3, GCOM1, CACNA2D1, GRM7, ADRB2, KCNH7, KCNIP4, GRIK2, CACNG2, KCNMA1, KCNG1

Synaptic plasticity	RIMS1, PTGER2, SLC24A2, NETO1, PTGS2

Vesicle exocytosis	PTPRN2, AMPH, RAB11B, SYNPR

Other	TPH2, CHRNA9, RIMBP2, ATXN1, CHRNB4, NOVA1, SNCAIP, CHRM3

In order to investigate how the GWAS-NR results compared with the joint APL tests and Fisher's tests, we examined the lists of top 5000 markers selected based on GWAS-NR, joint APL test and Fisher's test *P*-values. A total of 3328 of the markers are overlapped between the lists for the GWAS-NR and joint APL tests, while 1951 of the markers are overlapped between the lists for the GWAS-NR and Fisher's tests. Thus, GWAS-NR had a higher concordance with the joint APL tests than the Fisher's tests. The results suggested that Fisher's test may have the lowest sensitivity to identify the true positives, which is consistent with our simulation results. Moreover, 120 markers that are not overlapped between Illumina Infinium Human 1M beadchip and Illumina HumanHap550 BeadChip were among the top 5000 markers selected based on GWAS-NR. Some of the 120 markers are in the significant genes identified by haplotype blocks such as PUM2, A2BP1, DNER and SEMA4D.

In order to similarly investigate the overlap of candidate genes indentified by GWAS-NR and joint APL tests, we repeated the haplotype block scoring method with the top 5000 markers as identified by joint APL: this analysis resulted in 1924 significant LD blocks. Of these, 1257 overlapped with the blocks selected by GWAS-NR analysis. Identification of the RefSeq genes within with these 1257 shared regions showed that 380 potential candidate genes were shared by the two methods. In addition, GWAS-NR analysis produced 53 non-overlapping genes while the joint APL analysis produced 349 non-overlapping genes.

As GWAS-NR amplifies association signals that are replicated in multiple flanking markers and across data sets, the method can be expected to produce a reduced list of higher confidence candidate regions for follow-up, compared with standard single-locus methods. At the same time, GWAS-NR does not generate a large number of significant candidates in regions that would otherwise be ranked as insignificant. While it is not possible to exclude a role in autism for the 349 additional candidate genes produced by the joint APL analysis, it is notable that among the top 20 gene ontology pathways reported by DAVID [41-43] for this set of genes, not one is specific to neuronal function (data not shown). This analysis highlights the utility of GWAS-NR to narrow and prioritize follow-up gene lists.

## Discussion

We propose the use of GWAS-NR, a noise-reduction method for genome-wide association studies which aims to enhance the power to detect true positive associations for follow-up analysis. Our results demonstrate that GWAS-NR is a powerful method for the enhancement of the detection of genetic associations. Simulation evidence using a variety of disease models indicates that, when markers are ranked by *P*-values and candidates are selected based on a threshold rank, GWAS-NR achieves higher classification rates than the use of joint *P*-values or Fisher's method. In simulated data, the GWAS-NR also achieves strong performance when there is imperfect marker overlap across datasets and when the closest disease-related polymorphism is not typed. As Müller-Myhsok and Abel have observed, when less-than-maximum LD exists between a disease locus and the closest biallelic marker, the required sample size to achieve a given level of power may increase dramatically, particularly if there is a substantial difference in allele frequencies at the disease marker and the analysed marker [49].

In the context of allelic association, noise can be viewed as observed but random association evidence (for example, false positives) that is not the result of true LD with a susceptibility or causative variant. Such noise is likely to confound studies of complex disorders, where genetic heterogeneity among affected individuals or complex interactions among multiple genes may result in modest association signals that are difficult to detect. The influence of positive noise components is also likely to contribute to the so-called 'winner's curse' phenomenon, whereby the estimated effect of a putatively associated marker is often exaggerated in the initial findings, compared with estimated effects in follow-up studies [50]. GWAS-NR appears to be a promising approach to address these challenges.

By amplifying signals in regions where association evidence is locally correlated across datasets, the GWAS-NR captures information that may be omitted or underutilized in single-marker analysis. However, the GWAS-NR can achieve no advantage over simple joint analysis when flanking markers provide no supplementary information. This is likely to be true when a true risk locus is typed directly and a single-marker association method is used or when a true risk haplotype is typed directly and the number of markers examined in a haplotype-based analysis is of the same length.

Joint analysis generally has more power than individual tests due to the increase of sample size. Therefore, GWAS-NR, which uses *P*-values from individual analyses as well as joint analysis of multiple datasets, is expected to have more power than individual tests. However, if there are subpopulations in the sample and the association is specific to a subpopulation, joint analysis may not be as powerful as an individual test for the subpopulation with the association signal. If samples from multiple populations are analysed jointly, test results for individual datasets should also be carefully examined with the GWAS-NR results.

It is common for linear filters to include a large set of estimated parameters to capture cross-correlations in the data at multiple leads and lags. However, in a genomic context, the potentially uneven spacing of markers and varying strength of linkage disequilibrium between markers encouraged us to apply a parsimonious representation that would be robust to data structure. We expect that a larger, well-regularized parameterization may enhance the performance of the noise filter, particularly if the filter is constructed to adapt to varying linkage disequilibrium across the genome. This is a subject of further research.

Our simulation results indicate that applying the modified TPM to select LD blocks based on GWAS-NR can have conservative type I error rates. The original TPM reported by Zaykin *et al. *[40] produced the expected level of type I error, as a known correlation matrix was used in the simulations to account for correlation among *P*-values due to LD among markers. However, the true correlation is unknown in real datasets. Accordingly, we estimated correlations in our simulations and analysis by bootstrapping replicates of samples, as well as using the sample correlation between *P*-values obtained though single marker APL and sliding window haplotype analysis. It is possible that the use of estimated correlations may introduce extra variations in the Monte-Carlo simulations of TPM, which may contribute to conservative type I error rates. As we have demonstrated that GWAS-NR achieves higher sensitivity at each level of specificity, the resulting regions with top rankings can be expected to be enriched for true associations when such associations are actually present in the data, even if the LD block selection procedure is conservative. Overall, the simulation results suggest that GWAS-NR can be expected to produce a condensed set of higher confidence follow-up regions, and that this prioritization strategy can control the number of false positives at or below the expected number in analysis.

### Autism

Our data identify potential candidate genes for autism that encode a large subset of proteins involved in the outgrowth and guidance of axons and dendrites to their appropriate synaptic targets. Our results also suggest secondary involvement of genes involved in synaptogenesis and neurotransmission which further contribute to the assembly and function of neural circuitry. Taken together, these findings augment existing genetic, epigenetic and neuropathological evidence suggestive of altered neurite morphology, cell migration, synaptogenesis and excitatory-inhibitory balance in autism [49].

Altered dendritic structure is among the most consistent neuroanatomical findings in autism [51,52] and several other neurodevelopmental syndromes including Down, Rett and fragile-X [53,54]. Recent neuroanatomical findings include evidence of subcortical, periventricular, hippocampal and cerebellar heterotopia [55] and altered microarchitecture of cortical minicolumns [56], suggestive of dysregulated neuronal migration and guidance. In recent years, evidence from neuroanatomical and neuroimaging studies has led a number of researchers to propose models of altered cortical networks in autism, emphasizing the possible disruption of long-range connectivity and a developmental bias toward the formation of short-range connections [57,58].

Neurite regulation is a common function of numerous top-ranking candidates. PUM2 codes for pumilio homolog 2, which regulates dendritic outgrowth, arborization, spine formation and filopodial extension of developing and mature neurons [59]. DNER regulates the morphogenesis of cerebellar Purkinje cells [60] and acts as an inhibitor to retinoic-acid induced neurite outgrowth [61]. A2BP1 binds with ATXN2 (SCA2), a dosage-sensitive regulator of actin filament formation that is suggested to mediate the loss of cytoskeleton-dependent dendritic structure [62]. SEMA4D induces axonal growth cone collapse [63] and promotes dendritic branching and complexity in later stages of development [64,65]. CDH8 regulates hippocampal mossy fibre axon fasciculation and targeting, complementing N-cadherin (CDH2) in the assembly of synaptic circuits [66].

**Figure 3:**
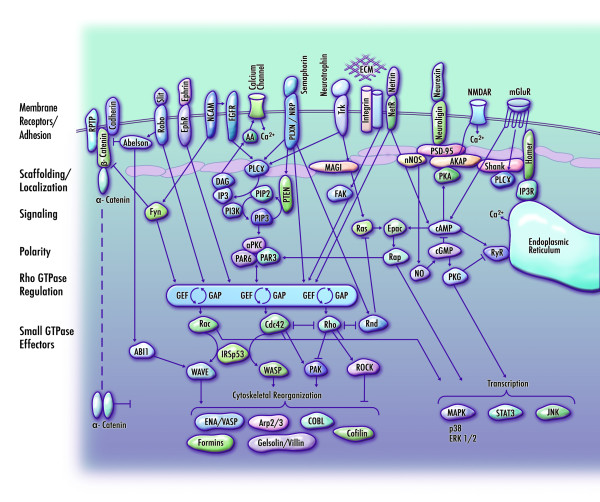
**Simplified schematic illustrating molecular mechanisms of neurite regulation**. Extracellular events such as cell contact [79], guidance cues [64], neurotransmitter release [80], and interactions with extracellular matrix components [46] are detected by receptors and cell adhesion molecules at the membrane surface and are transduced via cytoplasmic terminals and multidomain scaffolding proteins [47] to downstream signalling molecules [81-83]. Polarity and directional navigation is achieved by coordinating local calcium concentration [84], Src family kinases [85], cyclic nucleotide activation (cAMP and cGMP) [86], and phosphoinositide signalling molecules which affect the spatial distribution and membrane recruitment of proteins that regulate the neuronal cytoskeleton [87]. Chief among these regulators are the small Rho family GTPases RhoA, Rac and Cdc42, which serve as molecular 'switches' to activate downstream effectors of cytoskeletal remodelling [88]. In developed neurons, this pathway further regulates the formation of actin-dependent microarchitecture such as mushroom-like dendritic spines at the postsynaptic terminals of excitatory and inhibitory synapses [89]. This simplified schematic presents components in an exploded format for tractability, and includes an abridged set of interactions. Additional File 9 presents autism candidate genes identified by GWAS-NR having known roles in neurite regulation. RPTP (receptor protein tyrosine phosphatase); EphR (Eph receptor); FGFR (fibroblast growth factor receptor); EphR (Eph receptor); PLXN (plexin); NRP (neuropilin); Trk (neurotrophin receptor); ECM (extracellular matrix); NetR (netrin receptor); NMDAR (NMDA receptor); mGluR (metabotropic glutamate receptor); AA (arachidonic acid); PLCγ (phospholipase C, gamma); MAGI (membrane associated guanylate kinase homolog); IP3 (inositol 1,4,5-trisphosphate); DAG (diacylglycerol); PIP2 (phosphatidylinositol 4,5-bisphosphate); PIP3 (phosphatidylinositol 3,4,5-trisphosphate); PI3K (phosphoinositide-3-kinase); nNOS (neuronal nitric oxide synthase); NO (nitric oxide); IP3R (inositol trisphosphate receptor); RyR (ryanodine receptor); GEF (guanine exchange factor); GAP (GTPase activating protein); MAPK (mitogen-activated protein kinase); and JNK (c-Jun N-terminal kinase).

Neurite outgrowth and guidance can be conceptualized as a process whereby extracellular signals are transduced to cytoplasmic signalling molecules which, in turn, regulate membrane protrusion and neuronal growth cone navigation by reorganizing the architecture of the neuronal cytoskeleton. In general, neurite extension is dependent on microtubule organization, while the extension and retraction of finger-like filopodia and web-like lamellipodia from the neuronal growth cone is dependent on actin dynamics. Gordon-Weeks [67] and Bagnard [68] provide excellent overviews relating to growth cone regulation and axon guidance. Figure 3 provides a simplified overview of some of these molecular interactions.

The autism gene candidates identified by GWAS-NR show functional enrichment in processes, including adhesion, cell motility, axonogenesis, cell morphogenesis and neuron projection development. Notably, a recent analysis of rare CNVs in autism by the Autism Genome Project Consortium indicates similar functional enrichment in the processes of neuronal projection, motility, proliferation, and Rho/Ras GTPase signalling [21].

We propose that, in autism, these processes are not distinct functional classifications but instead cooperate as interacting parts of a coherent molecular pathway regulating the outgrowth and guidance of axons and dendrites. Consistent with this view, the candidate set is enriched for numerous binding domains commonly found in proteins that govern neuritogenesis. These include immunoglobulin, cadherin, pleckstrin homology, MAM, fibronectin type-III and protein tyrosine phosphatase (PTP) domains [69-71].

The cytoskeletal dynamics of extending neurites are largely governed by the activity of Rho-GTPases, which act as molecular switches to induce actin remodelling. Molecular evidence suggests that disassociation of catenin from cadherin promotes the activation of Rho-family GTPases Rac and Cdc42, resulting in cytoskeletal rearrangement [72]. Guanine nucleotide exchange factors (GEFs) such as DOCK1 [73] and KALRN [74] activate Rho-GTPases by exchanging bound guanosine diphosphate (GDP) for guanosine triphosphate (GTP), while GTPase activating proteins (GAPs) such as SRGAP3 [75] increase the rate of intrinsic GTP hydrolysis to inactivate GTPases. Pleckstrin homology domains, characteristic of several GEFs and GAPs, bind to phosphoinositides to establish membrane localization and also may play a signalling role in GTPase function [76]. Certain GTPases outside of the Rho family, particularly Rap and Ras, also exert an influence on cytoskeletal dynamics and neurite differentiation [77,76].

Several genes in the candidate set with established roles in neurite formation and guidance have been previously implicated in autism. These include A2BP1 (*P *= 3.60E-05), ROBO2 (2.00E-03), SEMA5A (2.30E-03), EN2 (4.00E-03), CACNA1G (6.00E-03), PTEN (8.00E-03), NRXN1 (1.10E-02), FUT9 (1.80E-02), DOCK8 (2.10E-02), NRP2 (2.60E-02) and CNTNAP2 (2.70E-02). Other previously reported autism candidate genes with suggestive roles in neurite regulation include PCDH9 (1.76E-03), CDH9 (6.00E-03) and CSMD3 (2.10E-02).

The enriched presence of transcription factors in the candidate set is intriguing, as many of these candidates, including CUX2, SIX3, MEIS2 and ZFHX1B have established roles in the specification of GABAergic cortical interneurons [76]. Many guidance mechanisms in the neuritogenic pathway, such as Slit-Robo, semaphorin-neuropilin, and CXCR4 signalling also direct the migration and regional patterning of interneurons during development. Proper targeting of interneurons is vital to the organization of cortical circuitry, including minicolumnar architecture which is reported to be altered in autism [78]. Thus, the functional roles of the candidate genes we identify may embrace additional forms of neuronal motility and targeting.

## Conclusions

We proposed a noise-reduction methodology, GWAS-NR, to enhance the ability to detect associations in GWAS data. By amplifying signals in regions where association evidence is locally correlated across datasets, the GWAS-NR captures information that may be omitted or underutilized in single-marker analysis. Simulation evidence demonstrates that under a variety of disease models, GWAS-NR achieves higher classification rates for true positive associations, compared with the use of joint p-values or Fisher's method.

The GWAS-NR method was applied to autism data, with the objective of prioritizing regions of association for follow-up analysis. Gene set analysis was conducted in order to examine if the identified autism candidate genes were over-represented in any biological pathway relative to the background genes. The significance of a given pathway suggests that the pathway may be associated with autism due to the enrichment of autism candidate genes in that pathway. We find that many of the implicated genes cooperate within a coherent molecular mechanism. This neuritogenic pathway regulates the transduction of membrane-associated signals to downstream cytoskeletal effectors that induce the directional protrusion of axons and dendrites. This mechanism provides a framework that embraces numerous genetic findings in autism to date, and is consistent with neuroanatomical evidence. While confirmation of this pathway will require additional evidence such as the identification of functional variants, our results suggest that autistic pathology may be mediated by the dynamic regulation of the neuronal cytoskeleton, with resulting alterations in dendritic and axonal connectivity.

## Abbreviations

ADI-R: Autism Diagnostic Interview - Revised; AGRE: Autism Genetic Resource Exchange; APL: association in the presence of linkage; AUC: area under the curve; CNV: copy number variation; DAVID: Database for Annotation, Visualization and Discovery; GTP: guanosine triphosphate; LD: linkage disequilibrium; GWAS: Genome-wide association studies; NR: noise reduction; RefSeq: Reference Sequence; ROC: receiver operating characteristic; SNP: single nucleotide polymorphism; TPM: truncated product method.

## Competing interests

The authors declare that they have no competing interests.

## Authors' contributions

All co-authors contributed to writing the manuscript. JPH was the primary author of the manuscript, developed the statistical methods and the design for their implementation and contributed pathway analysis of candidate genes. RHC contributed to the development of the statistical methods and study design and also conducted statistical analyses. AJG contributed to molecular analysis and interpretation. JMJ, DS and DM conducted statistical analyses. IK and PLW performed molecular analysis and interpretation. JMV performed molecular analysis and contributed to the study design. ERM provided input into study design, methods development, statistical analyses, and interpretation of findings. MLC analysed clinical data and contributed to the study design. JRG performed molecular analysis, interpreted data and contributed to the study design. JLH provided input to study design and statistical analyses. MPV contributed to the design of the study, development of methods, coordination of statistical and molecular analysis and interpretation of data. She is also the primary investigator on the parent study.

## Supplementary Material

Additional File 1 Appendix Click here for file

Additional File 2 **Table S7: Haplotype configuration **Association configuration for the power simulations. Click here for file

Additional File 3 **Comparative classification rates for genome-wide association studies - noise reduction (GWAS-NR), Joint analysis and Fisher's Test**. GWAS-NR has an area under the curve (AUC) of 0.679 and the joint and Fisher's tests have AUC of 0.624 and 0.604, respectively, for the recessive model. Also GWAS-NR has AUC of 0.855 and the joint and Fisher's tests have AUC of 0.781 and 0.751, respectively, for the multiplicative model. For the dominant model, AUC for GWAS-NR, the joint and Fisher's tests are 0.964, 0.871 and 0.853, respectively. For the additive model, AUC for GWAS-NR, the joint and Fisher's tests are 0.893, 0.806 and 0.771, respectively. Click here for file

Additional File 4 **Flow Chart: GWAS-NR analysis workflow in autism datasets**. A flow chart demonstrating the data analysis and candidate gene selection of the autism datasets presented. *HIHG: *Hussman Institute for Human Genomics dataset, *AGRE: *Autism Genetic Resource Exchange dataset, *APL: *Association in the Presence of Linkage, *GWAS-NR: *Genome-wide Association Study - Noise Reduction, *DAVID: *Database for Annotation, Visualization and Integrated Discovery. Click here for file

Additional File 5 **Table S1: linkage disequilibrium (LD) blocks identified by Genome-wide Association Study - Noise Reduction (GWAS-NR)**. *E*very LD block identified by GWAS-NR and haplotype analysis with a *P*-value < 0.05 is listed with the chromosome start and stop position, the length in basepairs of the LD block, and the minimum GWAS-NR *P*-value of the block. Click here for file

Additional File 6 **Table S2: RefSeq genes overlapping linkage disequilibrium (LD) blocks identified by Genome-wide Association Study - Noise Reduction (GWAS-NR)**. Every LD block identified by GWAS-NR and haplotype analysis with a *P*-value < 0.05 and that overlaps a gene in the RefSeq database is listed with the chromosome start and stop position, the length in basepairs of the LD block, the minimum GWAS-NR *P*-value of the block, and the RefSeq name of the gene(s) that overlap the block. Click here for file

Additional File 7 **Table S3: RefSeq genes nearest to linkage disequilibrium (LD) blocks identified by Genome-wide Association Study - Noise Reduction (GWAS-NR)**. Every LD block identified by GWAS-NR and haplotype analysis with a *P*-value < 0.05 that does not overlap with a gene in the reference sequence (RefSeq) database is listed with the chromosome start and stop position, the length in basepairs of the LD block, the minimum GWAS-NR *P*-value of the block and the RefSeq name of the gene(s) that is nearest to the block. Click here for file

Additional File 8 **Table S4: Autism candidate genes identified by Genome-wide Association Study - Noise Reduction (GWAS-NR)**. A complete list of reference sequence (RefSeq) genes either overlapping or nearest to every LD blocks with the *P*-value of either the overlapping or nearest block. Click here for file

Additional File 9 **Table S5: Autism candidate genes [Genome-wide Association Study - Noise Reduction (GWAS-NR)] having known roles in neurite outgrowth and guidance**. A list of autism candidate genes with known roles in neurite outgrowth and axon guidance followed by a comment on molecular function and PubMed identifications of supporting literature. Click here for file

Additional File 10 **Table S6: autism candidate genes [Genome-wide Association Study - Noise Reduction (GWAS-NR)] having suggestive roles in neurite outgrowth and guidance**. A list of autism candidate genes with presumptive roles in neurite outgrowth and axon guidance followed by a comment on molecular function and PubMed identifications of supporting literature. Click here for file

## References

[B1] Armitage P Test for linear trends in proportions and frequencies Biometrics 1955 11 375 386 10.2307/3001775

[B2] McCarthy MI Abecasis GR Cardon LR Goldstein DB Little J Ioannidis JP Hirschhorn JN Genome-wide association studies for complex traits: consensus, uncertainty and challenges Nat Rev Genet 2008 9 356 369 10.1038/nrg2344 18398418

[B3] Zaykin DV Westfall PH Young SS Karnoub MA Wagner MJ Ehm MG Testing association of statistically inferred haplotypes with discrete and continuous traits in samples of unrelated individuals Hum Hered 2002 53 79 91 10.1159/000057986 12037407

[B4] Tzeng JY Wang CH Kao JT Hsiao CK Regression-based association analysis with clustered haplotypes through use of genotypes Am J Hum Genet 2006 78 231 242 10.1086/500025 16365833 PMC1380232

[B5] Sha Q Chen HS Zhang S A new association test using haplotype similarity Genet Epidemiol 2007 31 577 593 10.1002/gepi.20230 17443704

[B6] Xiong M Zhao J Boerwinkle E Generalized T2 test for genome association studies Am J Hum Genet 2002 70 1257 1268 10.1086/340392 11923914 PMC447600

[B7] Rakovski CS Xu X Lazarus R Blacker D Laird NM A new multimarker test for family-based association studies Genet Epidemiol 2007 31 9 17 10.1002/gepi.20186 17086514

[B8] Akey J Jin L Xiong M Haplotypes vs single marker linkage disequilibrium tests: what do we gain? Eur J Hum Genet 2001 9 291 300 10.1038/sj.ejhg.5200619 11313774

[B9] Fan R Knapp M Genome association studies of complex diseases by case-control designs Am J Hum Genet 2003 72 850 868 10.1086/373966 12647259 PMC1180349

[B10] Grant SF Qu HQ Bradfield JP Marchand L Kim CE Glessner JT Grabs R Taback SP Frackelton EC Eckert AW DCCT/EDIC Research Group Follow-up analysis of genome-wide association data identifies novel loci for type 1 diabetes Diabetes 2009 58 290 295 10.2337/db08-1022 18840781 PMC2606889

[B11] International Multiple Sclerosis Genetics Consortium (IMSGC) Comprehensive follow-up of the first genome-wide association study of multiple sclerosis identifies KIF21B and TMEM39A as susceptibility loci Hum Mol Genet 2010 19 953 962 10.1093/hmg/ddp542 20007504 PMC2816610

[B12] Steffenburg S Gillberg C Hellgren L Andersson L Gillberg IC Jakobsson G Bohman M A twin study of autism in Denmark, Finland, Iceland, Norway, and Sweden J Child Psychol Psychiatry 1989 30 405 416 10.1111/j.1469-7610.1989.tb00254.x 2745591

[B13] Bailey A Le Couteur A Gottesman I Bolton P Simonoff E Yuzda E Rutter M Autism as a strongly genetic disorder: evidence from a British twin study Psychol Med 1995 25 63 77 10.1017/S0033291700028099 7792363

[B14] Bolton P Macdonald H Pickles A Rios P Goode S Crowson M Bailey A Rutter M A case-control family history study of autism J Child Psychol Psychiatry Allied Disciplines 1994 35 877 900 10.1111/j.1469-7610.1994.tb02300.x 7962246

[B15] American Psychiatric Association Diagnostic and Statistical Manual of Mental Disorders (DSM-IV), Text Revision 2000 Washington, DC: American Psychiatric Press

[B16] Jamain S Quach H Betancur C Rastam M Colineaux C Gillberg IC Soderstrom H Giros B Leboyer M Gillberg C Autism Research International Sibpair Study Mutations of the X-linked genes encoding neuroligins NLGN3 and NLGN4 are associated with autism Nat Genet 2003 34 27 29 10.1038/ng1136 12669065 PMC1925054

[B17] Marshall CR Noor A Vincent JB Lionel AC Feuk L Skaug J Shago M Moessner R Pinto D Ren Y Structural variation of chromosomes in autism spectrum disorder Am J Hum Genet 2008 82 477 488 10.1016/j.ajhg.2007.12.009 18252227 PMC2426913

[B18] Wang K Zhang H Ma D Bucan M Glessner JT Abrahams BS Salyakina D Imielinski M Bradfield JP Sleiman PM Common genetic variants on 5p14.1 associate with autism spectrum disorders Nature 2009 459 528 533 10.1038/nature07999 19404256 PMC2943511

[B19] Ma D Salyakina D Jaworski JM Konidari I Whitehead PL Andersen AN Hoffman JD Slifer SH Hedges DJ Cukier HN A genome-wide association study of autism reveals a common novel risk locus at 5p14.1 Ann Hum Genet 2009 73 263 273 10.1111/j.1469-1809.2009.00523.x 19456320 PMC2918410

[B20] Weiss LA Arking DE Gene Discovery Project of Johns Hopkins & the Autism Consortium Daly MJ Chakravarti A A genome-wide linkage and association scan reveals novel loci for autism Nature 2009 461 802 808 10.1038/nature08490 19812673 PMC2772655

[B21] Pinto D Pagnamenta AT Klei L Anney R Merico D Regan R Conroy J Magalhaes TR Correia C Abrahams BS Functional impact of global rare copy number variation in autism spectrum disorders Nature 2010 2010 466 7304 368 372 10.1038/nature09146 PMC3021798 20531469

[B22] Durand CM Betancur C Boeckers TM Bockmann J Chaste P Fauchereau F Nygren G Rastam M Gillberg IC Anckarsater H Mutations in the gene encoding the synaptic scaffolding protein SHANK3 are associated with autism spectrum disorders Nat Genet 2007 39 25 27 10.1038/ng1933 17173049 PMC2082049

[B23] Goffin A Hoefsloot LH Bosgoed E Swillen A Fryns JP PTEN mutation in a family with Cowden syndrome and autism Am J Med Genet 2001 105 521 524 10.1002/ajmg.1477 11496368

[B24] Serajee FJ Nabi R Zhong H Mahbubul Huq AH Association of INPP1, PIK3CG, and TSC2 gene variants with autistic disorder: implications for phosphatidylinositol signalling in autism J Med Genet 2003 40 e119 10.1136/jmg.40.11.e119 14627686 PMC1735327

[B25] Folstein SE Rosen-Sheidley B Genetics of autism: complex aetiology for a heterogeneous disorder Nat Rev Genet 2001 2 943 955 10.1038/35103559 11733747

[B26] Bacchelli E Blasi F Biondolillo M Lamb JA Bonora E Barnby G Parr J Beyer KS Klauck SM Poustka A International Molecular Genetic Study of Autism Consortium (IMGSAC) Screening of nine candidate genes for autism on chromosome 2q reveals rare nonsynonymous variants in the cAMP-GEFII gene Mol Psychiatry 2003 8 916 924 10.1038/sj.mp.4001340 14593429

[B27] Abuhatzira L Shemer R Razin A MeCP2 involvement in the regulation of neuronal alpha-tubulin production Hum Mol Genet 2009 18 1415 1423 10.1093/hmg/ddp048 19174478

[B28] Gharani N Benayed R Mancuso V Brzustowicz LM Millonig JH Association of the homeobox transcription factor, ENGRAILED 2, 3, with autism spectrum disorder Mol Psychiatry 2004 9 474 484 10.1038/sj.mp.4001498 15024396

[B29] Barnby G Abbott A Sykes N Morris A Weeks DE Mott R Lamb J Bailey AJ Monaco AP Candidate-gene screening and association analysis at the autism-susceptibility locus on chromosome 16p: evidence of association at GRIN2A and ABAT Am J Hum Genet 2005 76 950 966 10.1086/430454 15830322 PMC1196454

[B30] Collins AL Ma D Whitehead PL Martin ER Wright HH Abramson RK Hussman JP Haines JL Cuccaro ML Gilbert JR Pericak-Vance MA Investigation of autism and GABA receptor subunit genes in multiple ethnic groups Neurogenetics 2006 7 167 174 10.1007/s10048-006-0045-1 16770606 PMC1513515

[B31] Roeder K Wasserman L Genome-wide significance levels and weighted hypothesis testing Stat Sci 2009 24 398 413 10.1214/09-STS289 20711421 PMC2920568

[B32] de Cheveigne A Simon JZ Sensor noise suppression J Neurosci Methods 2008 168 195 202 10.1016/j.jneumeth.2007.09.012 17963844 PMC2253211

[B33] Edwards TL Bush WS Turner SD Dudek SM Torstenson ES Schmidt M Martin E Ritchie MD Generating Linkage Disequilibrium Patterns in Data Simulations using genomeSIMLA Lect Notes Comput Sci 2008 4973 24 35 full_text19779634

[B34] Chung RH Hauser ER Martin ER The APL test: extension to general nuclear families and haplotypes and the examination of its robustness Hum Hered 2006 61 189 199 10.1159/000094774 16877866

[B35] AGRE http://www.agre.org/

[B36] Sparrow SS Balla D Cicchetti D Vineland Adaptive Behavior Scales 1984 MN: AGS 10.1093/jpepsy/10.2.215 4020603

[B37] Sparrow SS Cicchetti DV Balla D Vineland Adaptive Behavior Scales 2005 2 MN: AGS 10.1093/jpepsy/10.2.215 4020603

[B38] Patterson N Price AL Reich D Population structure and eigenanalysis PLoS Genet 2006 2 e190 10.1371/journal.pgen.0020190 17194218 PMC1713260

[B39] Gabriel SB Schaffner SF Nguyen H Moore JM Roy J Blumenstiel B Higgins J DeFelice M Lochner A Faggart M The structure of haplotype blocks in the human genome Science 2002 296 2225 2229 10.1126/science.1069424 12029063

[B40] Zaykin DV Zhivotovsky LA Westfall PH Weir BS Truncated product method for combining *P*-values Genet Epidemiol 2002 22 170 185 10.1002/gepi.0042 11788962

[B41] Huang da W Sherman BT Lempicki RA Systematic and integrative analysis of large gene lists using DAVID bioinformatics resources Nat Protoc 2009 4 44 57 10.1038/nprot.2008.211 19131956

[B42] Dennis G Jr Sherman BT Hosack DA Yang J Gao W Lane HC Lempicki RA DAVID: Database for Annotation, Visualization, and Integrated Discovery Genome Biol 2003 4 P3 10.1186/gb-2003-4-5-p3 12734009 PMC193660

[B43] DAVID Bioinformatics Resources 6.7 http://david.abcc.ncifcrf.gov/

[B44] Tu IP Whittemore AS Power of association and linkage tests when the disease alleles are unobserved Am J Human Genetics 1999 64 641 649 10.1086/302253 9973303 PMC1377775

[B45] Galaxy http://www.main.g2.bx.psu.edu/

[B46] Andrews MR Czvitkovich S Dassie E Vogelaar CF Faissner A Blits B Gage FH French-Constant C Fawcett JW Alpha9 integrin promotes neurite outgrowth on tenascin-C and enhances sensory axon regeneration J Neurosci 2009 29 5546 5557 10.1523/JNEUROSCI.0759-09.2009 19403822 PMC6665849

[B47] Vessey JP Karra D More than just synaptic building blocks: scaffolding proteins of the post-synaptic density regulate dendritic patterning J Neurochem 2007 102 324 332 10.1111/j.1471-4159.2007.04662.x 17596209

[B48] Ashburner M Ball CA Blake JA Botstein D Butler H Cherry JM Davis AP Dolinski K Dwight SS Eppig JT Gene ontology: Tool for the unification of biology. the gene ontology consortium Nat Genet 2000 5 25 29 10802651 10.1038/75556 PMC3037419

[B49] Muller-Myhsok B Abel L Genetic analysis of complex diseases Science 1997 275 1328 9 author reply 1329-30 9064791

[B50] Kraft P Curses--winner's and otherwise--in genetic epidemiology Epidemiology 2008 19 649 51 discussion 657-8 10.1097/EDE.0b013e318181b865 18703928

[B51] Persico AM Bourgeron T Searching for ways out of the autism maze: genetic, epigenetic and environmental clues Trends Neurosci 2006 29 349 358 10.1016/j.tins.2006.05.010 16808981

[B52] Bauman ML Kemper TL Neuroanatomic observations of the brain in autism: a review and future directions Int J Dev Neurosci 2005 23 183 187 10.1016/j.ijdevneu.2004.09.006 15749244

[B53] Raymond GV Bauman ML Kemper TL Hippocampus in autism: a Golgi analysis Acta Neuropathol 1996 91 117 119 10.1007/s004010050401 8773156

[B54] Kaufmann WE Moser HW Dendritic anomalies in disorders associated with mental retardation Cereb Cortex 2000 10 981 991 10.1093/cercor/10.10.981 11007549

[B55] Kaufmann WE MacDonald SM Altamura CR Dendritic cytoskeletal protein expression in mental retardation: an immunohistochemical study of the neocortex in Rett syndrome Cereb Cortex 2000 10 992 1004 10.1093/cercor/10.10.992 11007550

[B56] Wegiel J Kuchna I Nowicki K Imaki H Wegiel J Marchi E Ma SY Chauhan A Chauhan V Bobrowicz TW The neuropathology of autism: defects of neurogenesis and neuronal migration, and dysplastic changes Acta Neuropathol 2010 119 755 770 10.1007/s00401-010-0655-4 20198484 PMC2869041

[B57] Casanova MF Buxhoeveden DP Switala AE Roy E Minicolumnar pathology in autism Neurology 2002 58 428 432 11839843 10.1212/wnl.58.3.428

[B58] Casanova M Trippe J Radial cytoarchitecture and patterns of cortical connectivity in autism Philos Trans R Soc Lond B Biol Sci 2009 364 1433 1436 10.1098/rstb.2008.0331 19528027 PMC2677589

[B59] Minshew NJ Keller TA The nature of brain dysfunction in autism: functional brain imaging studies Curr Opin Neurol 2010 23 124 130 10.1097/WCO.0b013e32833782d4 20154614 PMC2975255

[B60] Vessey JP Schoderboeck L Gingl E Luzi E Riefler J Di Leva F Karra D Thomas S Kiebler MA Macchi P Mammalian Pumilio 2 regulates dendrite morphogenesis and synaptic function Proc Natl Acad Sci USA 2010 107 3222 3227 10.1073/pnas.0907128107 20133610 PMC2840302

[B61] Maeda N Fukazawa N Ishii M Chondroitin sulfate proteoglycans in neural development and plasticity Front Biosci 2010 15 626 644 10.2741/3637 20036837

[B62] Fukazawa N Yokoyama S Eiraku M Kengaku M Maeda N Receptor type protein tyrosine phosphatase zeta-pleiotrophin signaling controls endocytic trafficking of DNER that regulates neuritogenesis Mol Cell Biol 2008 28 4494 4506 10.1128/MCB.00074-08 18474614 PMC2447117

[B63] Satterfield TF Jackson SM Pallanck LJ A *Drosophila *homolog of the polyglutamine disease gene SCA2 is a dosage-sensitive regulator of actin filament formation Genetics 2002 162 1687 1702 12524342 10.1093/genetics/162.4.1687 PMC1462369

[B64] Swiercz JM Worzfeld T Offermanns S Semaphorin 4D signaling requires the recruitment of phospholipase C gamma into the plexin-B1 receptor complex Mol Cell Biol 2009 29 6321 6334 10.1128/MCB.00103-09 19805522 PMC2786695

[B65] Vodrazka P Korostylev A Hirschberg A Swiercz JM Worzfeld T Deng S Fazzari P Tamagnone L Offermanns S Kuner R The semaphorin 4D-plexin-B signalling complex regulates dendritic and axonal complexity in developing neurons via diverse pathways Eur J Neurosci 2009 30 1193 1208 10.1111/j.1460-9568.2009.06934.x 19788569

[B66] Bekirov IH Nagy V Svoronos A Huntley GW Benson DL Cadherin-8 and N-cadherin differentially regulate pre- and postsynaptic development of the hippocampal mossy fiber pathway Hippocampus 2008 18 349 363 10.1002/hipo.20395 18064706 PMC2727457

[B67] Gordon-Weeks PR Neuronal Growth Cones 2000 Cambridge; New York: Cambridge University Press

[B68] Bagnard D Axon Growth and Guidance 2007 New York; Texas: Springer Science and Business Media; Landes Bioscience

[B69] Doherty P Walsh FS CAM-FGF receptor interactions: a model for axonal growth Mol Cell Neurosci 1996 8 99 111 10.1006/mcne.1996.0049 8954625

[B70] Sallee JL Wittchen ES Burridge K Regulation of cell adhesion by protein-tyrosine phosphatases: II. Cell-cell adhesion J Biol Chem 2006 281 16189 16192 10.1074/jbc.R600003200 16497667

[B71] Nakamura F Tanaka M Takahashi T Kalb RG Strittmatter SM Neuropilin-1 extracellular domains mediate semaphorin D/III-induced growth cone collapse Neuron 1998 21 1093 1100 10.1016/S0896-6273(00)80626-1 9856464

[B72] Noren NK Liu BP Burridge K Kreft B p120 catenin regulates the actin cytoskeleton via Rho family GTPases J Cell Biol 2000 150 567 580 10.1083/jcb.150.3.567 10931868 PMC2175185

[B73] Cote JF Motoyama AB Bush JA Vuori K A novel and evolutionarily conserved PtdIns(3,4,5)P3-binding domain is necessary for DOCK180 signalling Nat Cell Biol 2005 7 797 807 10.1038/ncb1280 16025104 PMC1352170

[B74] Xie Z Cahill ME Penzes P Kalirin loss results in cortical morphological alterations Mol Cell Neurosci 2010 43 81 89 10.1016/j.mcn.2009.09.006 19800004 PMC2818244

[B75] Yang Y Marcello M Endris V Saffrich R Fischer R Trendelenburg MF Sprengel R Rappold G MEGAP impedes cell migration via regulating actin and microtubule dynamics and focal complex formation Exp Cell Res 2006 312 2379 2393 10.1016/j.yexcr.2006.04.001 16730001

[B76] Schwamborn JC Puschel AW The sequential activity of the GTPases Rap1B and Cdc42 determines neuronal polarity Nat Neurosci 2004 7 923 929 10.1038/nn1295 15286792

[B77] Liu C Takahashi M Li Y Song S Dillon TJ Shinde U Stork PJ Ras is required for the cyclic AMP-dependent activation of Rap1 via Epac2 Mol Cell Biol 2008 28 7109 7125 10.1128/MCB.01060-08 18824540 PMC2593374

[B78] Hernandez-Miranda LR Parnavelas JG Chiara F Molecules and mechanisms involved in the generation and migration of cortical interneurons ASN Neuro 2010 2 e00031 10.1042/AN20090053 20360946 PMC2847827

[B79] Boscher C Mege RM Cadherin-11 interacts with the FGF receptor and induces neurite outgrowth through associated downstream signalling Cell Signal 2008 20 1061 1072 10.1016/j.cellsig.2008.01.008 18302981

[B80] Georgiev D Taniura H Kambe Y Takarada T Yoneda Y A critical importance of polyamine site in NMDA receptors for neurite outgrowth and fasciculation at early stages of P19 neuronal differentiation Exp Cell Res 2008 314 2603 2617 10.1016/j.yexcr.2008.06.009 18586028

[B81] Williams EJ Furness J Walsh FS Doherty P Activation of the FGF receptor underlies neurite outgrowth stimulated by L1, N-CAM, and N-cadherin Neuron 1994 13 583 594 10.1016/0896-6273(94)90027-2 7917292

[B82] Falk J Bonnon C Girault JA Faivre-Sarrailh C F3/contactin, a neuronal cell adhesion molecule implicated in axogenesis and myelination Biol Cell 2002 94 327 334 10.1016/S0248-4900(02)00006-0 12500940

[B83] Lin X Ogiya M Takahara M Yamaguchi W Furuyama T Tanaka H Tohyama M Inagaki S Sema4D-plexin-B1 implicated in regulation of dendritic spine density through RhoA/ROCK pathway Neurosci Lett 2007 428 1 6 10.1016/j.neulet.2007.09.045 17950529

[B84] Akiyama H Matsu-ura T Mikoshiba K Kamiguchi H Control of neuronal growth cone navigation by asymmetric inositol 1,4,5-trisphosphate signals Sci Signal 2009 2 ra34 10.1126/scisignal.2000196 19602704

[B85] Robles E Woo S Gomez TM Src-dependent tyrosine phosphorylation at the tips of growth cone filopodia promotes extension J Neurosci 2005 25 7669 7681 10.1523/JNEUROSCI.2680-05.2005 16107653 PMC6725397

[B86] Nishiyama M Hoshino A Tsai L Henley JR Goshima Y Tessier-Lavigne M Poo MM Hong K Cyclic AMP/GMP-dependent modulation of Ca2+ channels sets the polarity of nerve growth-cone turning Nature 2003 423 990 995 10.1038/nature01751 12827203

[B87] Dawes AT Edelstein-Keshet L Phosphoinositides and Rho proteins spatially regulate actin polymerization to initiate and maintain directed movement in a one-dimensional model of a motile cell Biophys J 2007 92 744 768 10.1529/biophysj.106.090514 17098793 PMC1779977

[B88] Govek EE Newey SE Van Aelst L The role of the Rho GTPases in neuronal development Genes Dev 2005 19 1 49 10.1101/gad.1256405 15630019

[B89] Calabrese B Wilson MS Halpain S Development and regulation of dendritic spine synapses Physiology (Bethesda) 2006 21 38 47 16443821 10.1152/physiol.00042.2005

