# Supplementary material for: A noise-reduction GWAS analysis implicates altered regulation of neurite outgrowth and guidance in autism
Source: Mol Autism. 2011 Jan 19;2:1. doi: 10.1186/2040-2392-2-1 (PMC3035032; doi:10.1186/2040-2392-2-1)
Supplement: Additional File 1 — Appendix [file 2040-2392-2-1-S1.DOC]

Appendix

***Simulations for performance comparisons between GWAS-NR and other methods***

We used genomeSIMLA [33] to simulate LD structures based on the Affymetrix 5.0 chip. To compare the performance of different methods, the first 5000 markers on chromosome 1 were simulated in two independent datasets, each with 500 complete parent-child trios. We selected a haplotype block with three markers as disease loci in the 5000 markers. The three-marker haplotypes had frequencies 0.521, 0.196, 0.217, and 0.066 for haplotypes 111, 121, 122, and 221, respectively. The haplotype configuration for association with the disease is shown in Additional File 10. Markers 1, 2, and 3 in the haplotypes have LD (*r*^2^) of 0.74, 0.18 and 0.05 with the disease locus, respectively. We simulated dominant, multiplicative, additive, and recessive disease models. The disease locus had a minor allele frequency of 0.2 and relative risk of 2. We assumed the disease had a prevalence of 0.005.

Two scenarios were tested. In the first scenario (Scen 1), we performed the sliding-window haplotype association in the presence of linkage (APL) [34] tests on the two independent datasets with a window size of 3. The sliding window haplotype tests also were performed on the joint dataset. In the second scenario (Scen 2), we assumed the two independent datasets were genotyped on different platforms and 80% of the markers overlapped for the multiplicative and recessive disease models and 50% of the markers overlapped for the dominant and additive models. The sliding window haplotype tests also were performed for the individual and joint datasets. For markers that were missing in one dataset, we used the p-values that were present in the other dataset as the p-values for the joint tests.

Additionally, we also simulated a haplotype block with 2 markers as disease haplotypes (Scen 3). The two-marker haplotypes had frequencies 0.521, 0.413 and 0.066 for haplotypes 11, 12, and 22, respectively. The haplotype configuration for association with the disease is also shown in Supplementary Table 1. Markers 1 and 2 in the haplotypes have LD (*r*^2^) of 0.52 and 0.07 with the disease locus, respectively. Dominant, multiplicative, additive and recessive models were also simulated. The sliding window haplotype tests also were performed on the two independent datasets and the joint dataset.

***Type I error Simulations for the modified TPM***

To evaluate the type I error rate for the modified TPM based on GWAS-NR, we simulated chromosome 1 with 19006 markers. LD blocks were identified using Gabriel's method [39] provided in PLINK. A total of 7651 LD blocks were identified. We simulated two independent datasets, each with 500 complete parent-child trios. The same procedure used to identify significant LD blocks in the autism datasets was applied to the simulated datasets. We performed GWAS-NR based on single-marker APL p-values from the two independent datasets and the joint dataset. We also performed GWAS-NR on p-values obtained from sliding-window haplotype tests with a haplotype length of three markers for the two datasets and the joint dataset. The minimum values (MIN_NR) of the GWAS-NR p-values obtained from single-marker tests, and the GWAS-NR p-values obtained from tests of three-marker haplotypes, were selected. The modified TPM was then applied to the 7651 LD blocks based on the MIN_NR for the markers in the blocks. Type I error rates were calculated based on the modified TPM p-values for the 7651 LD blocks.

Two scenarios were also simulated. In the first scenario, we assumed that the two independent datasets had the same genotyped markers. In the second scenario, we assumed that the two independent datasets were genotyped on different platforms and the second dataset had 50% of markers overlapped with the markers in the first dataset.
