# Supplementary material for: A noise-reduction GWAS analysis implicates altered regulation of neurite outgrowth and guidance in autism
Source: Mol Autism. 2011 Jan 19;2:1. doi: 10.1186/2040-2392-2-1 (PMC3035032; doi:10.1186/2040-2392-2-1)
Supplement: Additional File 2 — Table S7: Haplotype configuration Association configuration for the power simulations. [file 2040-2392-2-1-S2.DOC]

Additional Table 7 Haplotype configuration

| Haplotypes | P(H|D)^1^ | P(H|d)^2^ |
| --- | --- | --- |
| Three-marker haplotypes | | |
| 111 | 0 | 0.521 |
| 121 | 0 | 0.196 |
| 122 | 0 | 0.217 |
| 221 | 1 | 0.066 |
| Two-marker haplotypes | | |
| 11 | 0.2 | 0.521 |
| 12 | 0 | 0.414 |
| 22 | 0.8 | 0.066 |

^1^Haplotype frequencies in the presence of the disease allele

^2^Haplotype frequencies in the presence of the wild-type allele
