# Supplementary figures and images for: A noise-reduction GWAS analysis implicates altered regulation of neurite outgrowth and guidance in autism
Source: Mol Autism. 2011 Jan 19;2:1. doi: 10.1186/2040-2392-2-1 (PMC3035032; doi:10.1186/2040-2392-2-1)

| 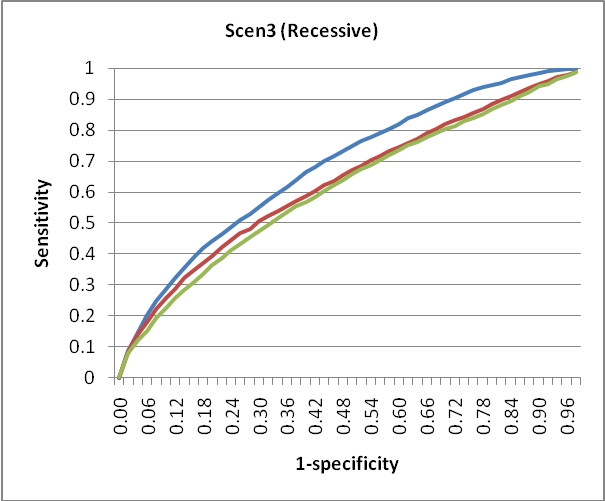 | 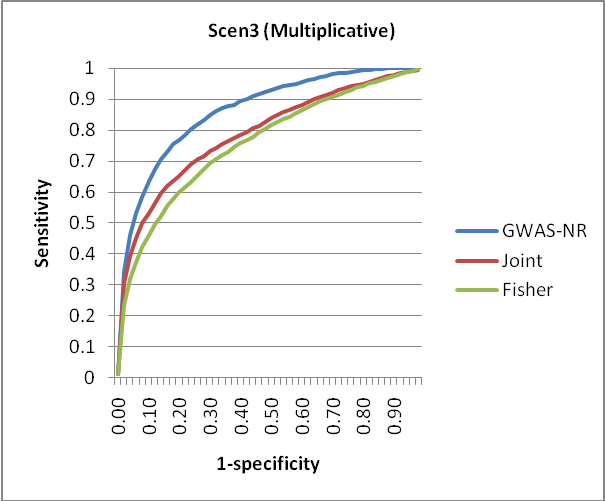 |
| --- | --- |
| 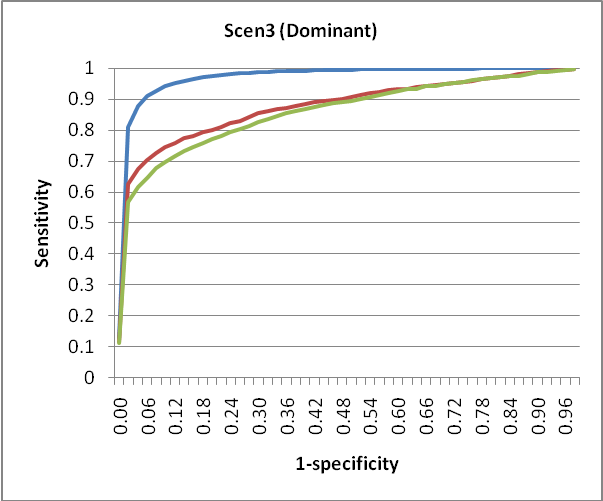 | 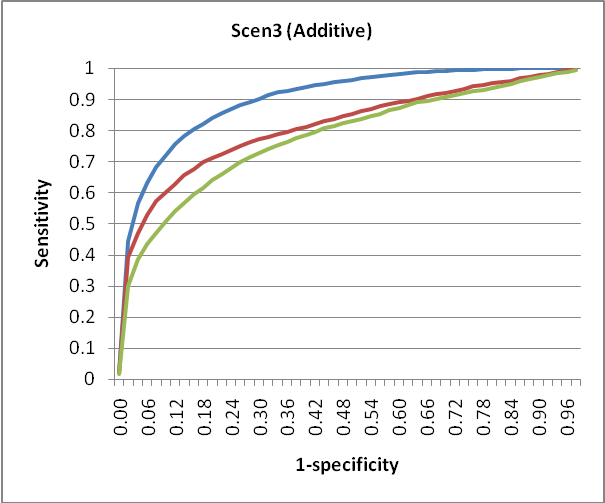 |

Supplement: Additional File 3 — Comparative classification rates for genome-wide association studies - noise reduction (GWAS-NR), Joint analysis and Fisher's Test. GWAS-NR has an area under the curve (AUC) of 0.679 and the joint and Fisher's tests have AUC of 0.624 and 0.604, respectively, for the recessive model. Also GWAS-NR has AUC of 0.855 and the joint and Fisher's tests have AUC of 0.781 and 0.751, respectively, for the multiplicative model. For the dominant model, AUC for GWAS-NR, the joint and Fisher's tests are 0.964, 0.871 and 0.853, respectively. For the additive model, AUC for GWAS-NR, the joint and Fisher's tests are 0.893, 0.806 and 0.771, respectively. [file 2040-2392-2-1-S3.DOC]

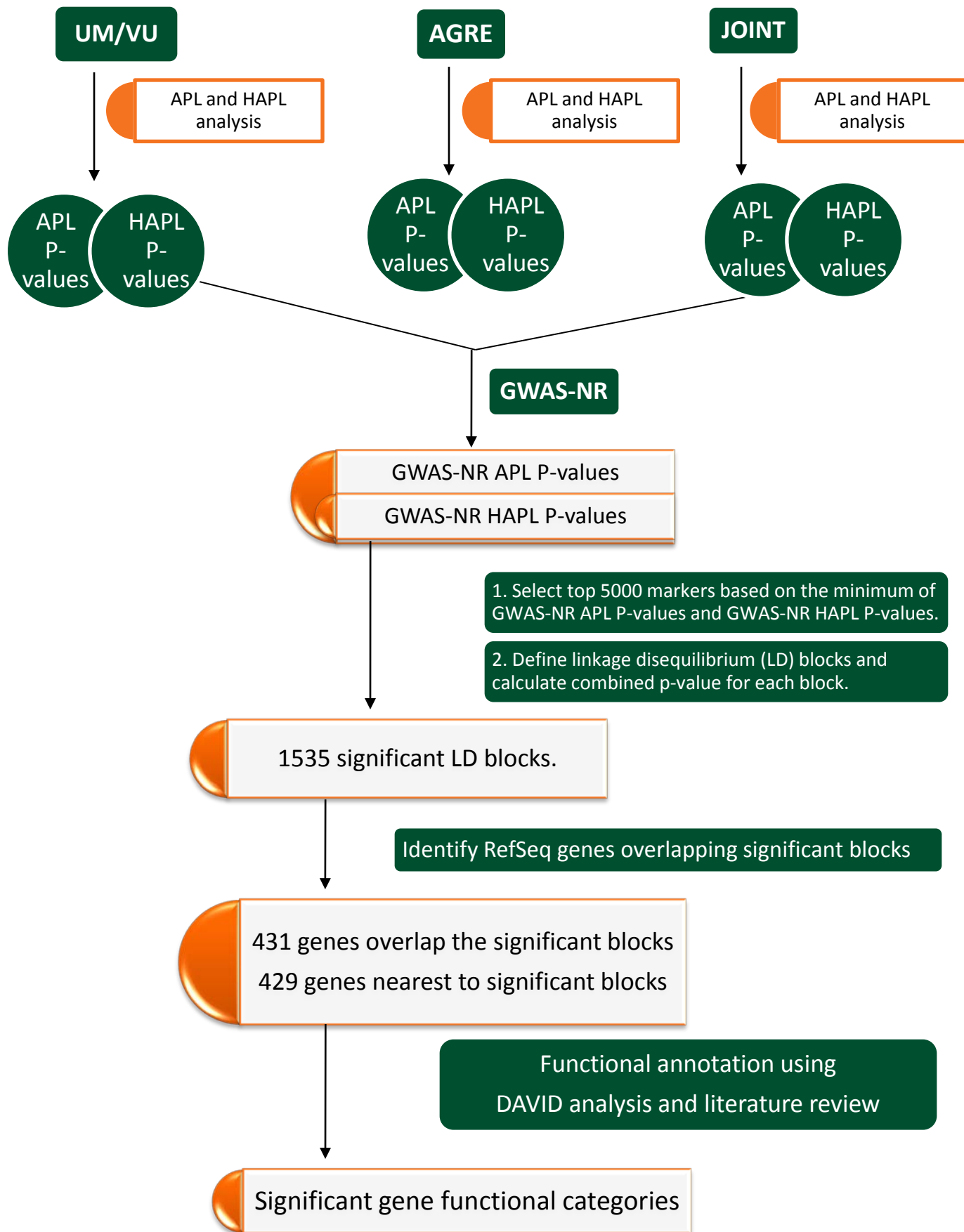

Supplement: Additional File 4 — Flow Chart: GWAS-NR analysis workflow in autism datasets. A flow chart demonstrating the data analysis and candidate gene selection of the autism datasets presented. HIHG: Hussman Institute for Human Genomics dataset, AGRE: Autism Genetic Resource Exchange dataset, APL: Association in the Presence of Linkage, GWAS-NR: Genome-wide Association Study - Noise Reduction, DAVID: Database for Annotation, Visualization and Integrated Discovery. [file 2040-2392-2-1-S4.PDF]
